# Invasion away from roadsides was not driven by adaptation to grassland habitats in *Dittrichia graveolens* (stinkwort)

**DOI:** 10.1007/s10530-024-03359-6

**Published:** 2024-06-05

**Authors:** Miranda K. Melen, Emma D. Snyder, Michael Fernandez, Andrew Lopez, Nicky Lustenhouwer, Ingrid M. Parker

**Affiliations:** 1 Department of Ecology and Evolutionary Biology, University of California Santa Cruz, Santa Cruz, CA USA; 2 College of Natural & Applied Science, University of Guam, Mangilao, Guam USA; 3 School of Biological Sciences, University of Aberdeen, Aberdeen, UK

**Keywords:** Population spread, Invasion biology, Evolution, Adaptation, Plant, Asteraceae

## Abstract

Invasive plants along transportation corridors can significantly threaten ecosystems and biodiversity if they spread beyond anthropogenic environments. Rapid evolution may increase the ability of invading plant populations to establish in resident plant communities over time, posing a challenge to invasion risk assessment. We tested for adaptive differentiation in *Dittrichia graveolens* (stinkwort), an invasive species of ruderal habitat in California that is increasingly spreading away from roadsides into more established vegetation. We collected seeds from eight pairs of vegetated sites and their nearest (presumed progenitor) roadside population. We assessed differentiation between populations in roadside and vegetated habitat for germination behavior and for response to competition in a greenhouse experiment. We also tested for increased performance in vegetated habitat with a grassland field experiment including a neighbor removal treatment. Germination rates were slightly reduced in seeds from vegetated sites, which may indicate lower seed viability. Otherwise, plants did not show consistent differences between the two habitat types. Competition strongly reduced performance of *D. graveolens* in both the greenhouse and in the field, but plants originating from vegetated sites did not show enhanced competitive ability. Our findings show no evidence of adaptive differentiation between *D. graveolens* populations from roadside and vegetated habitats to date, suggesting that invasiveness in grasslands has not been enhanced by rapid evolution in the 40 + years since this species was introduced to California. Evolutionary constraints or potentially high levels of gene flow at this small scale may limit adaptation to novel habitats along roadsides.

**Supplementary Information:**

The online version contains supplementary material available at 10.1007/s10530-024-03359-6.

## Introduction

Only a small proportion of introduced species will become invasive and have substantial ecological impacts (Williamson and Fitter 1996; Blackburn et al. 2011). Resource managers must allocate limited resources to management and eradication efforts focused on the most problematic species. Assessing the potential risk of newly introduced species is essential for prioritizing these efforts (Robinson et al. 2017). Such risk assessment includes evaluating which habitats are vulnerable to invasion by a species, and evaluating traits that make that species likely to invade those habitats (Diez et al. 2012; El-Barougy et al. 2021). Yet traits may evolve. In fact, introduced species have provided many classic examples of rapid evolution (Thompson 1998; Reznick et al. 2019). Rapid evolution of key traits may play a prominent role in promoting invasions (Maron et al. 2004; Buswell et al. 2011; Colautti and Barrett 2013; Turner et al. 2014). Evolutionary change is a key source of uncertainty in risk assessment for introduced species (Whitney and Gabler 2008; Clements and Ditommaso 2011), and there is a strong need for studies that will lead to a more comprehensive and nuanced understanding of where and when adaptive evolution promotes invasion.

Transportation corridors play an essential role in the early stages of invasion of introduced plants (Follak et al. 2018; Hogan et al. 2022). Vehicle traffic facilitates spread by moving plant propagules along roadways, accelerating dispersal rates, and establishing new roadside populations (Hansen and Clevenger 2005; Von Der Lippe and Kowarik 2007). Road construction and maintenance result in roadside soil compaction and erosion (Lázaro-Lobo and Ervin 2019; Mills et al. 2020). Runoff from roads increases salinity, chemical and heavy metal contaminants, and further contributes to soil erosion (Trombulak and Frissell 2000; Lázaro-Lobo and Ervin 2019). These roadside soil conditions provide ideal corridors for many stress- and disturbance-tolerant invasive plant species to take up residence and disperse because plant cover is lower (Mills et al. 2020) and plant competition pressures are reduced (Greenberg et al. 1997). However, an introduced plant must spread away from these anthropogenic environments to be considered a problematic invader. Here, roadside populations act as a source of propagule dispersal into adjacent plant communities (Hansen and Clevenger 2005; Kalwij et al. 2008; McDougall et al. 2011).

Life history theory and adaptive strategies could contribute to our understanding of the emergence of invasive species (Guo et al. 2018, 2022). Species that grow along roadsides exemplify the classic ruderal life history, with high fecundity, short generation time, and long-distance dispersal traits (Frenkel 1977; Dietz and Edwards 2006; Travis et al. 2009). Life history theory predicts that because of inherent evolutionary tradeoffs, ruderal species will be poor competitors in highly competitive habitats (Grime 1977; Burton et al. 2010; Pierce et al. 2017). In fact, Guo et al. (2022) found that species categorized as invasive were more associated with “competitor” traits while species categorized as naturalized but not invasive were associated with the “ruderal” traits. Yet individuals dispersing from a roadside population into more ecologically stable, vegetated areas will experience strong selection associated with greater competition and other environmental conditions such as higher soil fertility, differences in moisture availability, soil microbes (McDougall et al. 2011, 2018), and increased biotic interactions (e.g., herbivory) (Trombulak and Frissell 2000; Leblond et al. 2013, Muñoz et al. 2015). Evolution of traits conferring greater fitness in these vegetated habitats could increase invasiveness and exacerbate impact on competing resident species. For rapid evolution to promote invasion, however, selection would need to overcome those life history tradeoffs underlying adaptive strategies; we do not yet know how easily or how often this may occur. The first step is to look for evidence of divergence between populations actively spreading away from transportation corridors and their ruderal progenitors.

We used *Dittrichia graveolens* (L.) Greuter (stinkwort) as a model to investigate adaptive evolution’s role in promoting invasion away from roadside habitats. Introduced to California in the early 1980s, this herbaceous member of the Asteraceae was originally found in disturbed areas along railroad tracks and roads (Preston 1997; Brownsey et al. 2013a). Native to the Mediterranean Basin in Europe, *D. graveolens* grows in bare, disturbed habitats, including roadsides, crop and fallow land, stony riverbanks, and ruderal zones associated with annual or biennial weeds (Brullo and de Marco 2000; Rameau et al. 2008). It is a fall-flowering annual producing yellow radiate flowers and wind-dispersed fruits (Rameau et al. 2008). In California, *D. graveolens* germinates during the winter rainy season (Brownsey et al. 2013a) and spends several months growing vegetatively as a rosette before bolting in June. Flowering from September to December, *D. graveolens* sets seed and disperses from October through December (Brownsey 2012). Between its first observance in 1984 and 2012, *D. graveolens* spread to 62% of California counties (Brownsey et al. 2013a), reaching 79% of counties (46 out of 58) by 2020. It is now spreading east into the Sierra Nevada Mountains along transportation corridors (Calflora 2020).

More recently, *D. graveolens* in California has been observed spreading into areas with established vegetation (Brownsey et al. 2013a), including wildlands and rangelands (i.e., areas of natural vegetation grazed by livestock or wild herbivores). This calls attention to the potential invasion risk of *D. graveolens*. The USDA lists *D. graveolens* as a high-risk invasive species based on its high impact potential and ability to rapidly spread (USDA 2013). The plant is dangerous to livestock (Meadly 1965; Philbey and Morton 2000; Ponticelli et al. 2022) and causes contact dermatitis in humans (Thong et al. 2007; Ponticelli et al. 2022). In the County of Santa Clara, where the species was first observed, populations of *D. graveolens* can grow away from roadsides and co-occur with grassland species in established vegetative areas.

The introduction and spread of *D. graveolens* in California provides a unique opportunity to test the role of adaptive evolution in its spread away from roadsides. Earlier studies demonstrated rapid evolution in *D. graveolens* as it expanded its native range from the Mediterranean into higher latitudes (Lustenhouwer et al. 2018); in a common garden in the Netherlands, populations from the northern range edge flowered earlier, which increased fitness in the shorter growing season. In addition, niche modeling suggests that the species has expanded its climate niche since the mid-twentieth century, consistent with rapid evolutionary change (Lustenhouwer and Parker 2022). Similar to California, roadsides played a major role as transportation vectors during *D. graveolens’* native range expansion from the Mediterranean region to northern and central Europe (e.g., Brandes 2009; Frajman and Kaligarič 2009).

Here we studied whether *D. graveolens* populations in California have undergone evolution throughout their spread away from roads into more vegetated areas. We tested for phenotypic differences between paired populations: a population colonizing a vegetated area and its closest roadside, presumed progenitor, population. We quantified differences in germination behavior and used a greenhouse experiment to test for the response to competition in field soils. Finally, we used a field experiment in an established grassland to compare genotypes from roadside and vegetated sites in their phenology and response to release from competition.

## Methods and materials

### Study sites

The County of Santa Clara (37.36° N 121.97° W) is located at the southern end of the San Francisco Bay. The County encompasses the Santa Clara Valley, which is bounded by the Diablo Range to the east, Santa Cruz Mountains to the southwest, and San Francisco Bay salt marshes to the northwest. Due to its proximity to the Pacific Ocean and the moderating effects of the San Francisco Bay, the valley experiences a mild Mediterranean climate with warm, dry weather much of the year (Grossinger et al. 2007). The rainy season is predominantly from November to April and only yields about 375 mm of annual precipitation with a standard deviation of 125 mm (McKee et al. 2003).

### Plant community survey

**Fig. 1:**
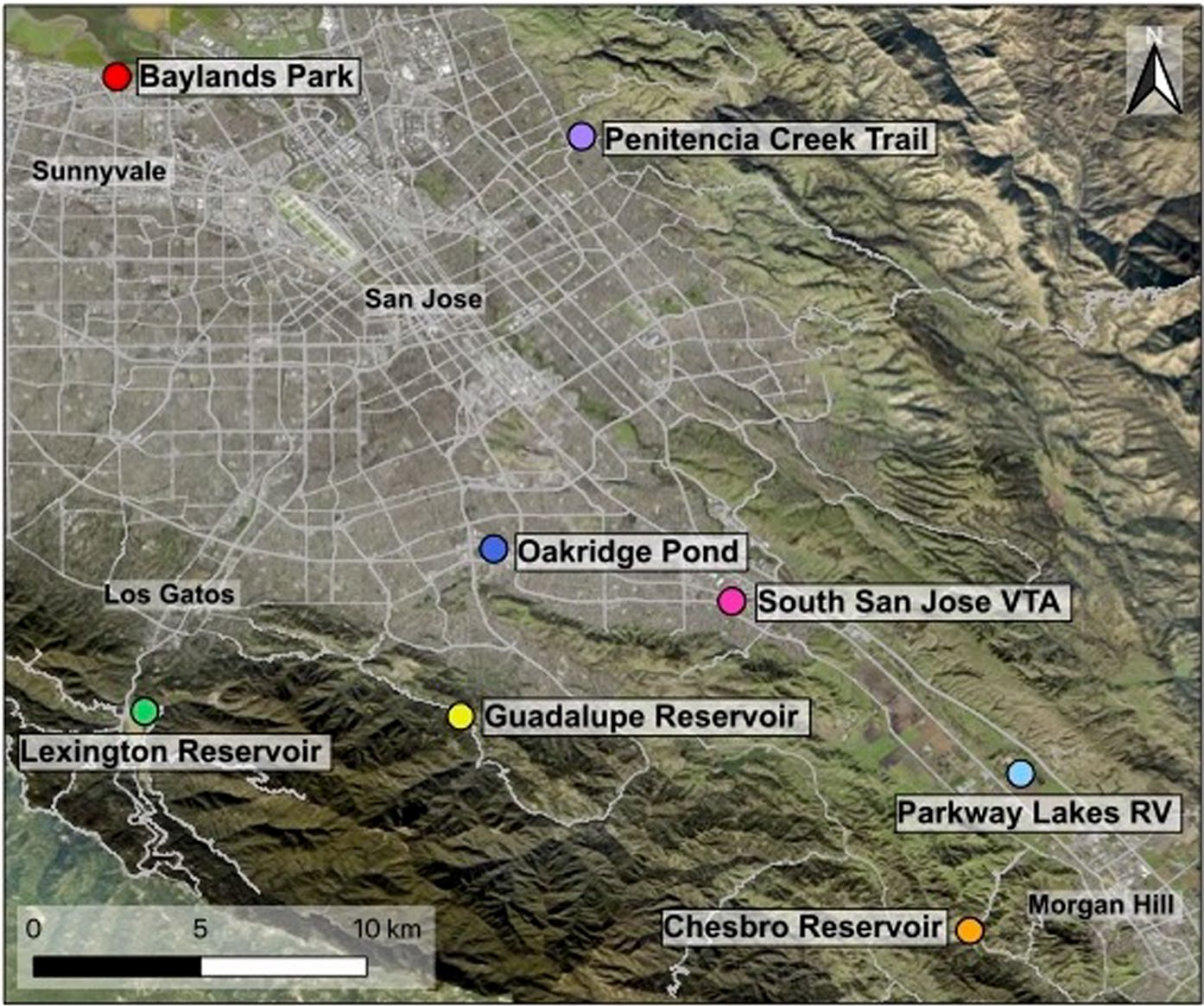
In September and October 2020, we collected *D. graveolens* seeds from eight sites in the County of Santa Clara. Each site had two paired populations: a population in a vegetated habitat and the closest roadside population. Map created using QGIS [3.32.0-Lima] (QGIS Development Team 2023)

In the summer of 2020, we identified *D. graveolens* populations in the County of Santa Clara within a 25-mile (~ 40 km) distance of the Alviso railway location where the species was originally found (Preston 1997). In collaboration with local resource managers and using online sources (e.g., CalFlora and Google Maps), we generated a list of populations where *D. graveolens* was growing in plant communities (vegetated habitat) at least 40 m from roadways. These vegetated habitats were not landscaped and generally associated with public parks or accessways that were dominated by common non-native annual species in the Poaceae and Asteraceae. Of an original list of 15 populations, our final study included 8 that were publicly accessible by foot and had not been eradicated before September 2020. For each population, we then located the nearest *D. graveolens* population along a roadside. We found roadside populations by walking away from the vegetated population along sidewalks and paths on the nearest hardened road. Each pair of populations in vegetated and roadside habitats is called a “site” (Fig. 1). We selected roads for this study that were hardened with an asphalt surface with speeds of 40.2–72.4 kph. The substrate of the roadside habitat was composed of engineered fill used in the construction of the roadbed. The two habitats (roadside and vegetated) within a site show strong spatial autocorrelation in many environmental characteristics (Table S1). Road density within a 3.14 km^2^ sampling area of each habitat ranged between 1 and 14 km/km^2^. Habitat elevation ranged between 3 and 210 m above mean sea level. Because primary spread of *D.* graveolens is along roads, and dispersal away from roadsides is a secondary process, we make the assumption that the nearest roadside population is the likely source of invasion for each vegetated population. The fact that the separate sites are far away from each other ensures that the populations in vegetated habitat are much more likely to be related to their nearest roadside than they are to each other.

Between July 1st and August 14th, 2020, we conducted plant community surveys at all 16 populations (Table S2). We walked the perimeter for each population of *D. graveolens* and placed pin flags around the edge. We then laid a 50 m transect tape along the longest axis (for roadsides, transects were always parallel to the road) and placed a 0.5 × 0.5 m quadrat at three equidistant points along the axis. We visually estimated percent cover within each quadrat for *D. graveolens,* other vegetation, and bare ground (sum equaling 100%). For each population, we identified species within the three quadrats and then walked the area to search for additional rare species. Taxa were identified to species when possible using The Jepson Manual: Vascular Plants of California (Second Edition).

In September and October of 2020, we sampled seeds from each of the 16 populations. We collected from at least 10 individuals, 3 m apart, for each population, along a randomly-placed transect. We combined seeds from all individuals in a population.

### Seed behavior

In the summer of 2021, we compared germination behavior of seeds from roadside and vegetated habitat types. We did three studies on different substrates: one on moist filter paper, one on engineered fill, and one on field topsoil collected from a site on the UC Santa Cruz campus. For this experiment, we used filter paper as a control to test seed behavior in ideal germination conditions, engineered fill as a proxy for roadside soils manufactured for roadbed construction, and field topsoil as a proxy for soils from plant communities. We germinated 50 seeds from each population in Petri dishes (80 Petri dishes; 5 replicates with 10 seeds each) for each substrate (filter paper, engineered fill, and field topsoil). Seeds were visually inspected beforehand to ensure that only fully developed seeds were used for all experiments. Petri dishes were sealed with Parafilm M™ and placed in a randomized block design in an incubation chamber with a daytime temperature of 23 °C from 0900 to 0100 h and a nighttime temperature of 19 °C from 0100 to 0900 h. We scored germination daily until no further germination was observed, then 7 more days (a total of 23 d on filter paper, 12 d on engineered fill, and 11 d on field topsoil). Signs of germination included the first emergence of the root radical or the cotyledon. Petri dishes were misted with DI water, and germinated seeds were removed once scored. We also took one homogenized sample of 30 seeds from each of the 16 populations and weighed them to the closest 0.001 g.

### Plant growth response to competition

To assess the response of *D. graveolens* to competition, and how it might have evolved during the invasion of vegetated sites, we exposed plants originating from roadside and vegetated habitats to a competition treatment in both a greenhouse and a field setting. The aim of the greenhouse experiment was to uncover genetic differentiation between roadside and vegetated habitats at high replication and highly controlled conditions. The field experiment (see *Relative fitness in a field setting*) was designed to look for adaptive differentiation under more realistic conditions.

We quantified response to competition in a greenhouse experiment with three treatments: *D. graveolens* grown alone (1 plant per pot), and *D. graveolens* with *Bromus hordeaceus* or with *Festuca perennis* (2 plants per pot). These non-native European annual grasses were selected because they are commonly found in California's annual grasslands (Seabloom et al. 2003; Dawson et al. 2007; HilleRisLambers et al. 2010) and were observed at or near the eight sites. We collected *B. hordeaceus* seeds from Blue Oak Ranch Reserve and *F. perennis* seeds from the Terrace Lands of Younger Lagoon Reserve on the UC Santa Cruz Coastal Science Campus.

We germinated *D. graveolens* seeds in the conditions described above. We germinated grasses in trays with potting mix and placed them under fluorescent light banks for 16-h length days and 8-h length nights. Once radicles and cotyledons emerged, seedlings were transplanted in sets of three (one for each treatment). We grew plants in D16 Deepots (5 cm diameter, 18 cm height) in the greenhouse using field topsoil collected from a UC Santa Cruz campus site. Pots were then randomized into a blocked design with each block consisting of one *D. graveolens* seedling from each of the 16 populations for each of the three competition treatments, N = 48 per block × 8 blocks (384 total).

After 4 months, we harvested *D. graveolens* aboveground biomass at the crown and dried it in a 60 °C oven for 3 days before weighing it.

### Relative fitness in a field setting

The field experiment was conducted at Blue Oak Ranch Reserve, part of the University of California Natural Reserve System. Blue Oak Ranch Reserve is located within the County of Santa Clara on the western slopes of Mount Hamilton in the Diablo Range, just east of San Jose, California, United States (37° 22′ 54.89ʺ N, 121° 44′ 10.55ʺ W). Blue Oak Ranch Reserve supported cattle grazing until 1972. This former rangeland represents a key habitat type threatened by the invasion of *D. graveolens*.

The experimental site is in a non-native grassland with a mixture of annual grasses and forbs (Table S3). Land managers mow the site in the spring. At the site, common herbivores include deer, rabbits, California ground squirrels, and wild pigs. We protected the experiment with hog fencing and reduced herbivory pressure from deer and rabbits. Although not near the experimental site, *D. graveolens* actively invades Blue Oak Ranch Reserve.

At Blue Oak Ranch Reserve, we tested whether rapid evolution during invasion into vegetated sites has enhanced fitness in the presence of grassland competitors. We established a 10 m × 26 m fenced field site and used a randomized block design with 10 blocks of 1.5 m^2^ plots. The data presented here are a subset of a larger ecological study elucidating *D. graveolens* response to different disturbance mechanisms. Here we focus on the response of plant genotypes and include only two treatments: grassland control (high competition) and complete competitor removal (no competition). We left the previous year’s thatch for the grassland control treatment and allowed resident vegetation (including the two species from our greenhouse experiment*, Bromus hordeaceus* and *Festuca perennis*, as well as 15 other plant species; Table S3) to grow throughout the experiment. For the competitor removal treatment, we tilled the soil to completely remove below and aboveground biomass in December 2020 and then weeded to remove aboveground biomass throughout the growing season.

In January 2021, we germinated seeds in Petri dishes in incubation chambers before transplanting them into soil collected in late December 2020 from Blue Oak Ranch Reserve. Seedlings grew in the greenhouse for about eight weeks until all plants had their first two true leaves emerge and lengthen. Seeds could not be sown directly into the field due to biosafety concerns.

We planted seedlings into each plot from February 27—March 24, 2021 (20 plots total). Each plot included one *D. graveolens* individual from each of the 16 populations, in a 4 × 4 grid centered on the plot. Plants were separated by 33 cm, with a 25 cm buffer. During the first month of growth, we replaced any *D. graveolens* that died. We surveyed plants weekly to assess *D. graveolens* survival and bud initiation until all plants had either produced buds or perished.

We terminated plants at the first sign of budding to prevent reproduction of a noxious weed. As proxies for reproductive output, we measured height and biomass. We harvested aboveground biomass by cutting at the root crown and drying in a 60 °C oven for three days before weighing. Height and biomass were strongly correlated (r = 0.74, N = 157), and results for the two response variables were similar. Therefore we present only the results for final biomass.

### Data analysis

We used *R* version 4.2.2 (2022–10-31; R Core Team 2022) for all statistical analyses. Our general approach for each response variable (except the plant community survey) was to run mixed effects models with, at minimum, a fixed effect for habitat (roadside vs. vegetated) and a random effect for site. The site random effect takes into account the genetic similarity between the two nearby populations within a site, and captures landscape-scale variation between sites in, for example, elevation and roadside density.

#### Plant community survey

We calculated the average percent cover of bare ground, *D. graveolens*, and other vegetation per population by taking the mean of the three quadrats along each transect. Species richness was the total number of species found at a population (the three quadrats + surrounding rare species survey). We evaluated differences in percent cover and species richness between source habitats (roadside and vegetated) using paired t-tests (N = 8 sites with pairs of roadside and vegetated populations at each site).

#### Seed behavior

We analyzed the germination rate on each of the three substrates (filter paper, engineered fill, and field topsoil) using a mixed-effects Cox proportional hazards model (coxme and survival packages; Therneau 2022a, b), with source habitat as a fixed effect and site, population, and dish number as nested random effects. We evaluated the main effect of source habitat using a Type II partial-likelihood-ratio test (car package; Fox and Weisberg 2019). We calculated average seed mass for each source habitat using a Welch Two Sample *t*-test.

#### Plant growth response to competition

We calculated response to competition as the log response ratio (LRR) of the aboveground biomass, LRR = ln (biomass with competitor/biomass alone), on a per-block basis (N = 8 blocks) for each of the 16 seed origins (vegetated or roadside habitat at each of the 8 sites). Therefore, each seed origin had 8 replicate LRR estimates for each competitor grass (*Bromus hordeaceus* and *Festuca perennis*). We fit a linear mixed effects model for each competitor with LRR as the response variable, source habitat as a fixed effect, and random effects for population nested in site, and block (lme4 package; Bates et al. 2015). Block was removed from the *B. hordeaceus* model because it did not explain sufficient variance, causing a singular fit. We tested for differences between source habitats using Type II Wald *F*-tests with Kenward-Rogers degrees of freedom (car package; Fox and Weisberg 2019). To evaluate whether each competitor grass affected the biomass of *D. graveolens*, we tested whether the LRR intercept was significantly different from zero using *t*-tests with Kenward-Rogers degrees of freedom (pbkrtest and lmerTest packages; Halekoh and Højsgaard 2014; Kuznetsova et al. 2017).

#### Relative fitness in a field setting

The field experiment had four response variables: survival (assessed both as total proportion surviving and time to death), final biomass at budding, and phenology (the survey date buds first appeared). We used a similar statistical approach for all response variables, fitting mixed effects models with source habitat, competition treatment, and their interaction as fixed effects; and initially including random effects for site, population nested in site, and block. Random effects that explained very low amounts of variance, causing singular fits, were removed. When interaction terms were not significant, they were removed and models were re-run with main effects only. Here we describe the structures of the final models.

We compared total survival to budding with a generalized linear mixed model using a binomial family with a logit link function; fixed effects were source habitat and competition treatment, and random effects were population nested in site, and block (glmmTMB package; Brooks et al. 2017). We evaluated the main effect of source habitat using a Type II Wald Chi-Square test (car package; Fox and Weisberg 2019). Second, we analyzed survival using a mixed-effects Cox proportional hazards model (coxme and survival packages; Therneau 2022a, b); fixed effects were source habitat and competition treatment, and random effects were population nested in site, and block. We evaluated the main effects of source habitat and competition treatment using likelihood ratio tests.

We analyzed final biomass at the time of bud production using a linear mixed effects model (lme4 package; Bates et al. 2015); fixed effects were source habitat, competition treatment, and their interaction, and the only remaining random effect was site. We evaluated the main and interaction effects using Type II Wald *F*-tests with Kenward-Rogers degrees of freedom (car package; Fox and Weisberg 2019). We used a log transformation of the biomass data to improve homoscedasticity.

To assess changes in phenology, we compared the timing to bud for those plants that reached the reproductive state, using a mixed-effects Cox proportional hazards model (coxme and survival packages; Therneau 2022a, b); fixed effects were source habitat and competition treatment, and random effects were population nested in site, and block. We evaluated the main effects using likelihood ratio tests.

## Results

### Plant community survey

**Fig. 2:**
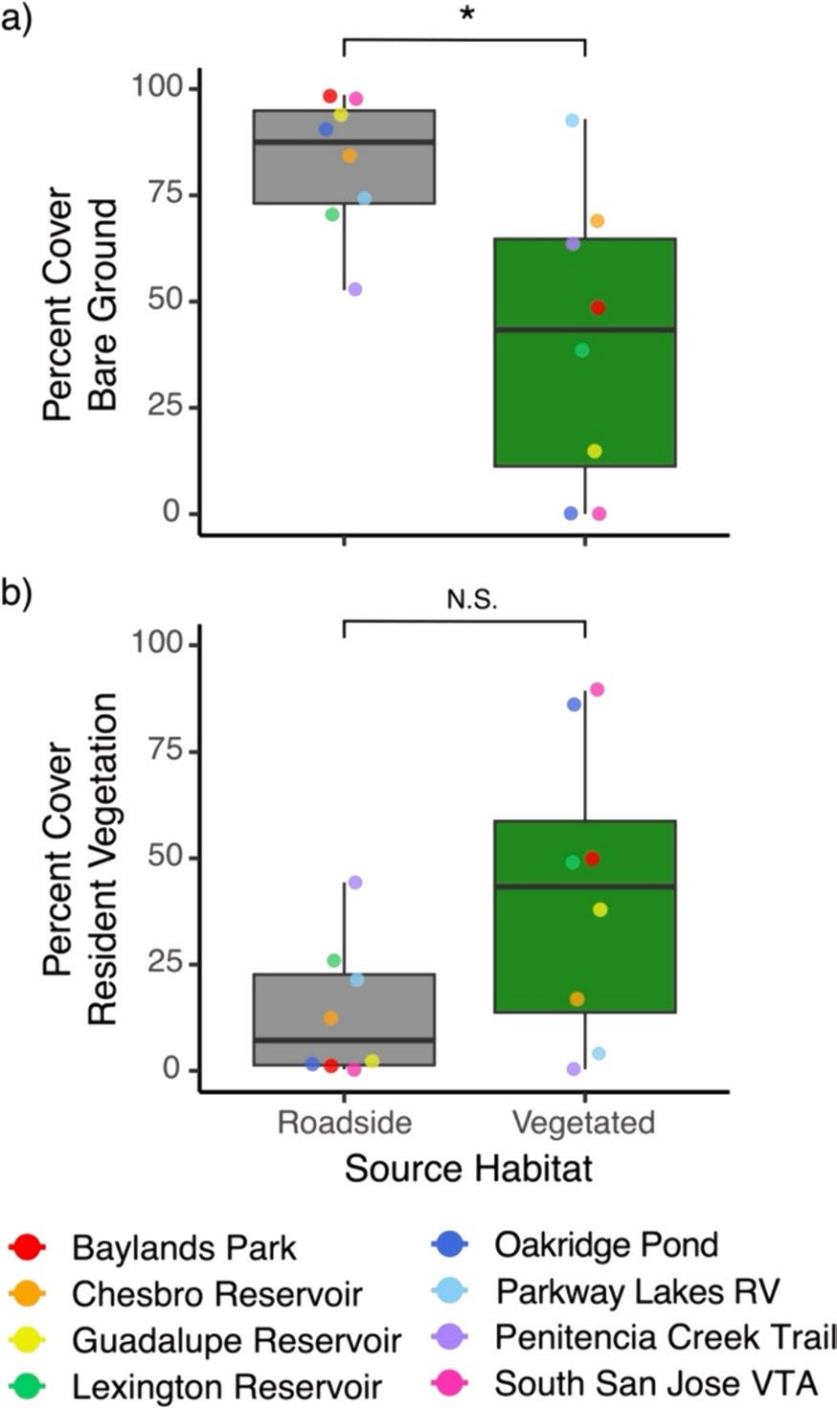
Differences in percent cover of bare ground (**a**) and resident vegetation (**b**) between roadside and vegetated sites (points indicate N = 8 sites per category). Boxes correspond to the median, first and third quartiles, and whiskers extend to the furthest value within 1.5 × the inter-quartile range. Star indicates significance of paired *t*-test

Roadside habitats had higher amounts of bare ground than vegetated habitats (mean difference 41.9%, 95% CI [4.2, 80], paired t_7_ = 2.63, *P* = 0.034; Fig. 2a). Roadside habitats appeared to have substantially less resident plant cover (not including *D. graveolens*) on average than vegetated habitats (Fig. 2b), but this difference was not significant (mean difference − 28%, 95% CI [− 67, 11], t_7_ = − 1.70, *P* = 0.13). Species richness was not significantly different (mean difference − 1.75, 95% CI [− 5, 1.5], t_7_ = 1.26, *P* = 0.25). Average species richness was 4.13 ± 2.59 SD at roadsides and 5.88 ± 3.27 SD at vegetated sites. Resident plant species at all sites were predominantly non-native annuals (Table S2).

### Seed behavior

**Fig. 3:**
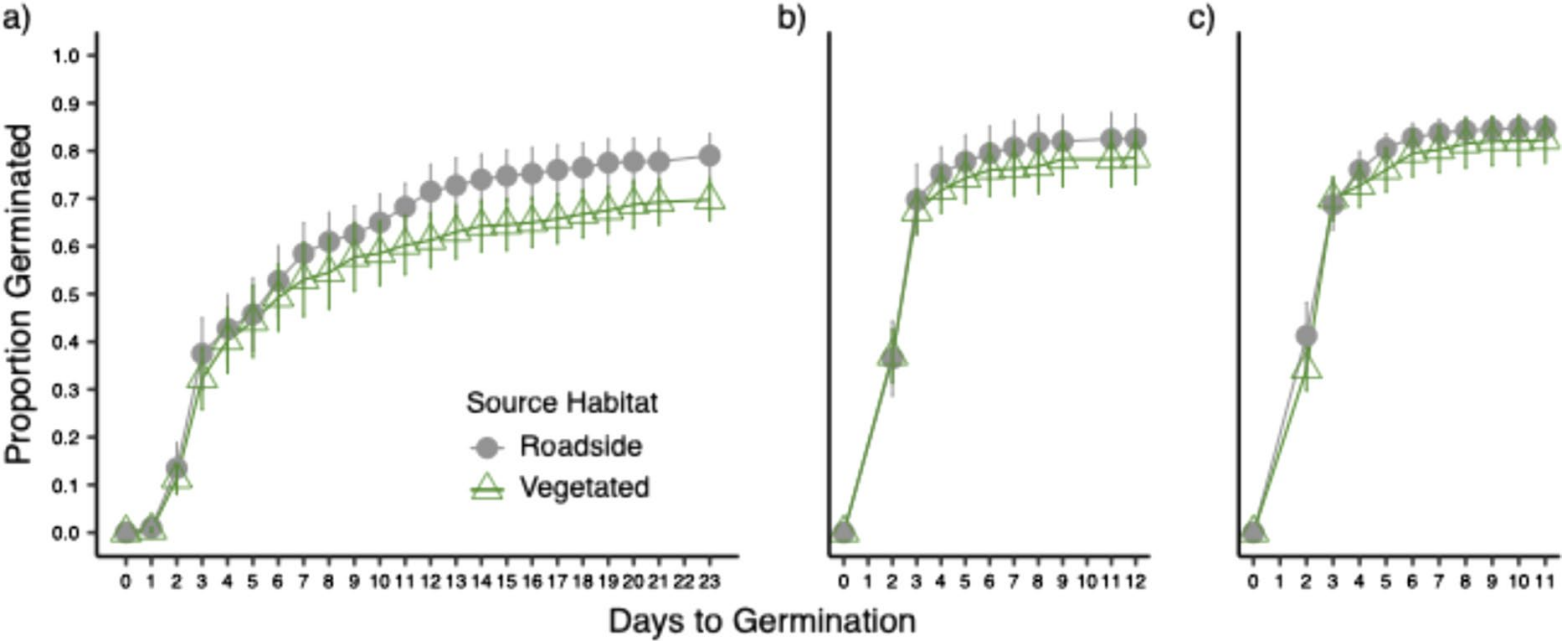
Cumulative proportion germinating per day of *D. graveolens* seeds collected from roadside (filled gray circles) and vegetated (open green triangles) source habitats. Seeds were germinated on (**a**) filter paper, (**b**) engineered fill, and (**c**) field topsoil. Values shown are means ± 1 SE of 8 sites, after first estimating site means from 5 dishes (proportion germinated out of 10 seeds each)

Seeds originating from vegetated habitats consistently had a slightly reduced probability of germination compared to seeds originating from roadside habitats (22% on filter paper, 11% on engineered fill, and 11% on field topsoil). This difference was significant on filter paper (relative risk of 0.78 ± 0.18 SE; X^2^_1_ = 85.60, *P* < 0.001; Fig. 3), engineered fill (relative risk of 0.89 ± 0.09 SE; X^2^_1_ = 80.86, *P* < 0.001), and field topsoil (relative risk of 0.89 ± 0.12 SE; X^2^_1_ = 30.6, *P* < 0.001). Average seed mass varied from 0.243 to 0.333 and did not differ between source habitats (roadside = 2.26 mg, vegetated = 2.37 mg; t_12.11_ = − 1.18, *P* = 0.259; Table S4).

### Plant growth response to competition

**Fig. 4:**
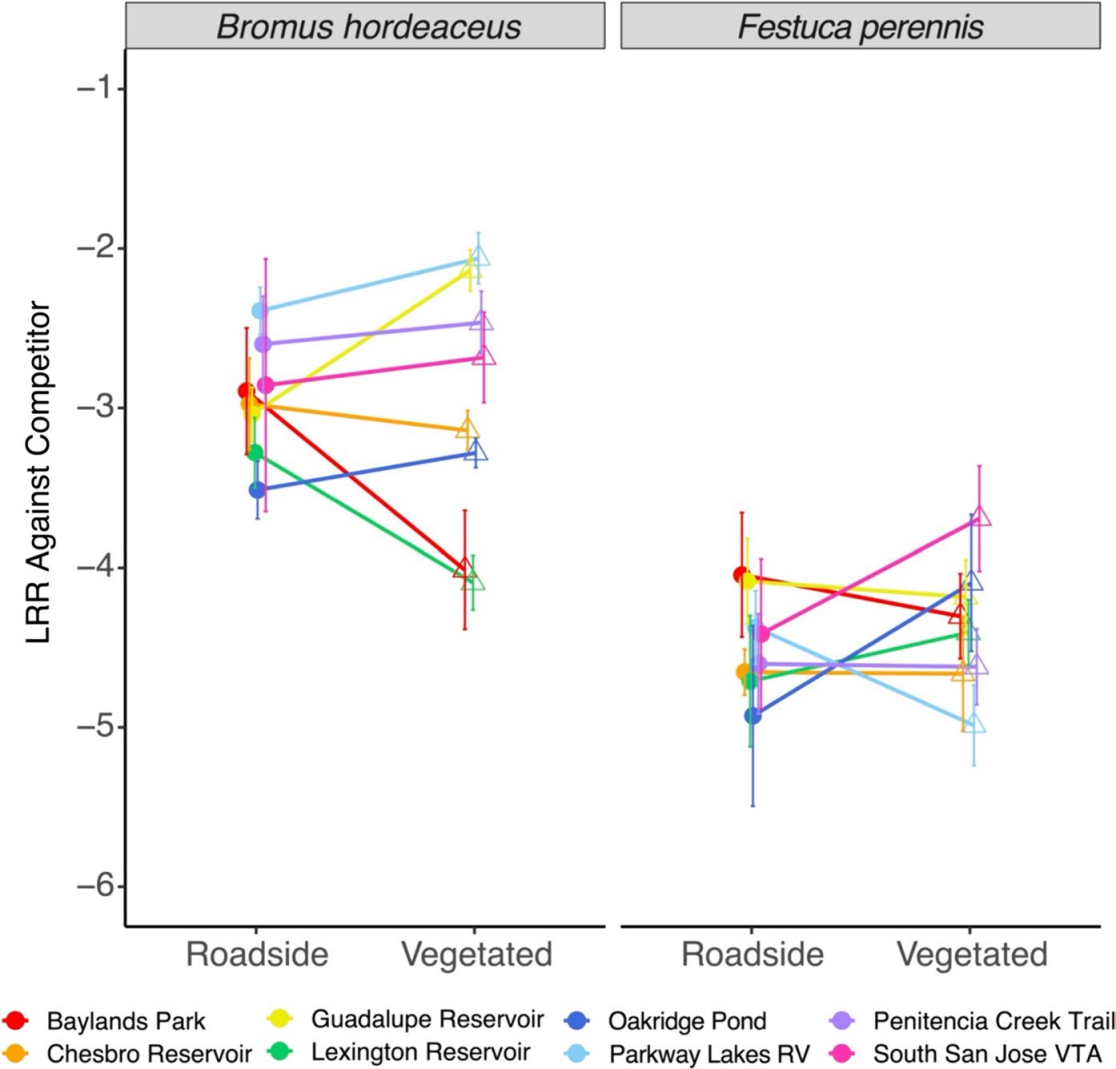
The log response ratio of biomass against each grass competitor (*B. hordeaceus* and *F. perennis*), calculated as the mean ± 1 SE across 8 replicate blocks for each seed origin. Lines of the same color connect seeds originating from paired roadside and vegetated habitats. Filled circles signify roadside habitats and open triangles signify vegetated habitats. We found that *D. graveolens* is a poor competitor, regardless of the source habitat

The growth of *D. graveolens* was strongly affected by competition with non-native grasses (Fig. 4). Both *Bromus hordeaceu*s (intercept = − 2.94 ± 0.22 SE, t_11.48_ = − 13.64, *P* < 0.001) and *Festuca perennis* (intercept = − 4.47 ± 0.13 SE, t_10.62_ = − 33.32, *P* < 0.001) strongly reduced the growth of *D. graveolens*. Plants from vegetated sites did not show evidence of a more robust response to competition: LRR did not differ between source habitats when *D. graveolens* was grown with either *B. hordeaceus* (F_1,7_ = 0.032, *P* = 0.86) or *F. perennis* (F_1,7_ = 0.37, *P* = 0.56).

### Relative fitness in a field setting

We found no significant interactions between source habitat and treatment for any of the response variables (overall survival: X^2^_1_ = 0.069, *P* = 0.79; survival analysis: X^2^_1_ = 0.018, *P* = 0.89; biomass: F_1,152.81_ = 2.34, *P* = 0.13, phenology: X^2^_1_ = 2.18, *P* = 0.14), indicating there was no differentiation between source habitats in their response to competition. Therefore the interactions were removed from the models.

**Fig. 5:**
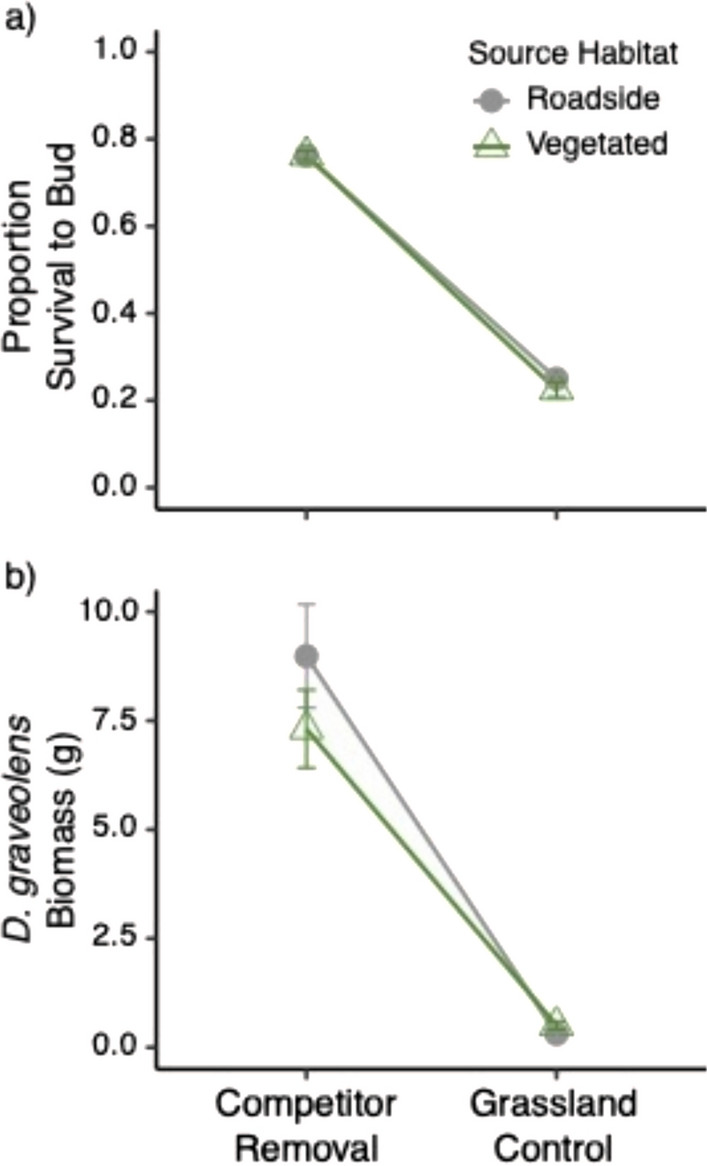
Plants from roadside and vegetated source habitats did not differ in fitness proxies survival and biomass between field treatments competitor removal and grassland (control) (Type II Wald Chi-Square test). (**a**) The proportion of *D. graveolens* that survived to produce buds (means ± 1 SE across 8 sites). (**b**) Aboveground biomass (g) of *D. graveolens* (means ± 1 SE of 8 sites, after first estimating site means from 10 plants). Filled gray circles signify roadside habitats and open green triangles signify vegetated habitats

We evaluated survival to reproduction in two ways. First, overall survival to reproduction was not affected by source habitat (X^2^_1_ = 0.069, *P* = 0.79), but was strongly affected by treatment (X^2^_1_ = 78.84, *P* < 0.001), with 53% greater survival to reproduction (absolute difference) in the competitor removal treatment compared to the grassland control. Second, consistent with the results for overall survival, our survival analysis showed that the competitor removal treatment reduced the mortality risk by 82% (X^2^_1_ = 99.09, *P* < 0.001; Fig. 5a). There was no significant difference between source habitats (X^2^_1_ = 0.0001, *P* = 0.99).

When we assessed aboveground biomass, we found that plants in the competitor removal treatment were significantly larger than those in the grassland control (F_1,152.42_ = 241.24, *P* < 0.0001; Fig. 5b). Similarly to survival, we found no significant difference between source habitats (F_1,151.85_ = 0.11, *P* = 0.74).

**Fig. 6:**
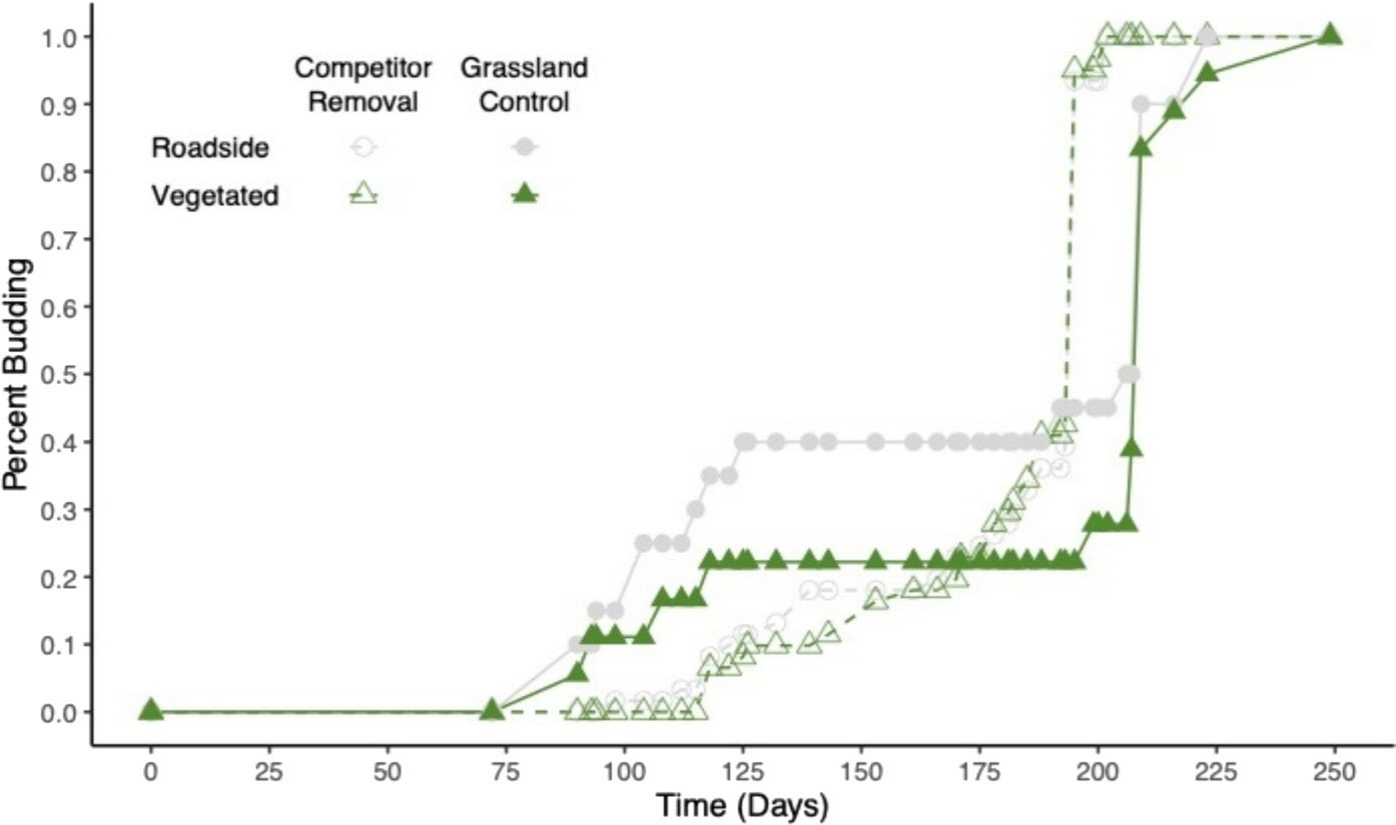
Flowering phenology (the percent of *D. graveolens* budding over time) showing roadside and vegetated source habitats for each treatment (competitor removal and grassland control). Gray lines signify roadside populations and green lines signify vegetated populations. Open symbols indicate competitor removal treatments and closed symbols indicate grassland control treatments

In terms of phenology, plants in the grassland control treatment initially started reproducing sooner, but by the end of the growing season, plants in the competitor removal treatment reproduced sooner on average than plants in the grassland control (X^2^_1_ = 56.13, *P* < 0.0001; Fig. 6). We found no significant difference between source habitats (X^2^_1_ = 0.29, *P* = 0.59).

## Discussion

Roads are vectors of invasion, as introduced species often spread along transportation corridors (Hansen and Clevenger 2005; Kalwij et al. 2008). However, to be considered invasive, a species must not just persist in ruderal populations but also spread aggressively away from roadsides, requiring traits that allow it to compete with resident plants. Evolution in introduced species can be an essential driver of invasion (Maron et al. 2004; Buswell et al. 2011; Colautti and Barrett 2013; Turner et al. 2014). *Dittrichia graveolens* is rapidly spreading along roads in California, and more recently has been observed establishing populations in vegetated areas away from roads. Does rapid evolution of competitive ability and other traits associated with surviving in vegetated habitats contribute to its invasiveness?

Our study found little evidence that populations of *D. graveolens* spreading away from roadsides into plant communities have evolved greater competitive ability. Response to competition for plant growth, survival, and reproduction did not differ between roadside and vegetated source habitats, and this was true in both greenhouse and field studies. Several factors could contribute to a lack of measurable adaptive differentiation between roadside versus vegetated populations. First, it may be that there has not been enough time for rapid evolution to occur. Introduced to California likely in the early 1980’s or late 1970’s, the annual *D. graveolen*s has spent around 40 generations in the County of Santa Clara where we studied it, although populations spreading away from roads may have experienced fewer generations in the competitive environment of the vegetated sites. Populations in California may ultimately evolve adaptations to vegetated environments away from roads in the future, but none have been detected so far. In contrast to our study, others have observed rapid evolution within a few decades of introduction. For example, Ethridge et al. (2023) found that *Setaria faberi* evolved larger leaf area within 34 generations as a result of agricultural selection pressure, and Dlugosch and Parker (2008b) found increased growth in *Hypericum canariense* from sites where introductions were < 25 generations. Moreover, Lustenhouwer et al. (2018) found rapid evolution of phenology in populations of our study species *D. graveolens* in the Netherlands within 2 decades of arrival in the country. These previous studies suggest that adaptive evolution in *D. graveolens* should have been possible within the time frame of its invasion in central California. However, evolutionary patterns can differ between traits of interest; Fletcher et al. (2023) found strong differentiation in biomass, height, and phenology between invasive populations of Johnsongrass (*Sorghum halepense*), but no difference in their response to competition as evaluated by growth on bare ground vs background vegetation.

Second, novel selection pressures may have been weak; selection may not differ substantially between the two habitat types. Our vegetated sites were often somewhat disturbed, and some were mowed; species composition was similar on and off the roadside (Figure S1), and plant diversity was low overall. Thus our vegetated sites might share some environmental conditions with roadsides in this suburban setting. However, roadside sites did show less resident plant cover and substantially more bare ground than vegetated sites (Fig. 2). We found that *D. graveolens* was strongly suppressed by competition in both the field experiments and the greenhouse experiment, and field experiments regardless of the competitor identity, suggesting competition should represent a strong selection pressure. In the greenhouse experiment, we saw that *D. graveolens* grew poorly in the presence of *B. hordeaceus* and *F. perennis*. This pattern was echoed in our field plot at Blue Oak Ranch Reserve, which was similar in structure and species composition to many of the 8 vegetated sites from which seeds were collected. The field experiment showed strong effects of competition from resident plants in the grassland on *D. graveolens* survival, phenology, and growth. Therefore, it is likely that selection on competitive ability does differ between roadsides and intact grasslands.

Variation among roadside locations and among vegetated locations would limit our ability to detect adaptive responses at individual sites, and this could contribute to the lack of consistent differences between habitats in our results. For example, an NMDS analysis showed substantial variation across the sites for plant composition (Figure S1). Perhaps most importantly, vegetated habitats varied substantially for overall resident plant cover and amount of bare ground across the sites (Fig. 2). At the time of our survey, two sites (Oakridge Pond and South San Jose VTA) showed over 80% cover of resident vegetation, while two sites (Parkway Lakes RV and Penitencia Creek Trail) had very low cover. In our greenhouse experiment, those two populations from vegetated habitats with high cover did show the predicted pattern of stronger competitive ability than their paired roadside populations. However, this anecdotal evidence was not supported by general trends.

Third, there may be a lack of heritable genetic variation for relevant traits, particularly for traits that increase competitive ability (Nei et al. 1975; Amos and Harwood 1998). The introduction of *D. graveolens* to California may have involved a significant reduction in genetic variation through a strong founder effect. The first observation of *D. graveolens* was in Alviso (San Jose) in 1984 (Preston 1997). This area, near the railway tracks, was also likely the first invasion point, with subsequent spread throughout the County of Santa Clara and eventually to much of California. Founder effects during invasion often reduce variation in invasive species (reviewed in Dlugosch and Parker 2008a; Dlugosch et al. 2015). However, many studies have shown evolutionary change despite reduced variation (Blows and Hoffmann 2005; Dlugosch and Parker 2008b; Estoup et al. 2016).

Finally, evolutionary divergence could be limited by gene flow from roadside to vegetated habitats (Ureta et al. 2008; Bagavathiannan et al. 2011). Gene flow is one of the primary factors counteracting local adaptation, and it is expected to have strong maladaptive (or swamping) effects in the relatively small populations of expanding range edges (May et al. 1975; Lenormand 2002; Anderson and Song 2020). Population pairs in our study ranged in distance from 540 m to as little as 40 m apart. Flowers can self-fertilize in *D. graveolens*, although flowers are also insect-pollinated in the native range (Rameau et al. 2008; Albaba 2015). Pollen dispersal distances and outcrossing rates have not been measured; however, McEvoy et al. (2023) found that heterozygosity is low across the genome, consistent with a highly self-fertilizing mating system. In contrast, seed dispersal is expected to be considerable in this wind-dispersed species with pappus-bearing seeds, suggesting high gene flow between populations is possible via seeds. Even with *D. graveolens*’ highly selfing mating system, gene flow over short distances could easily be why we did not observe adaptive evolution away from roadsides. This contrasts with other studies showing the evolutionary divergence of introduced species over more considerable distances (Colautti et al. 2009; Buswell et al. 2011; Clark 2018; Alexander and Levine 2019). Nonetheless, Fletcher et al. (2023) studied range-wide differentiation in invasive *Sorghum halepense* and still found no differentiation in competitive ability on the continental scale.

The only significant difference between roadside and vegetated source habitats was for germination success, which was lower overall in seeds from vegetated source habitats (Fig. 3). The higher proportion of ungerminated seeds from vegetated sites could indicate either lower seed viability or higher dormancy rates. Lower seed viability may reflect a poorer maternal environment or an increase in inbreeding and inbreeding depression in these nascent populations (Nei et al. 1975; Barrett and Husband 1990). Higher dormancy rates could be adaptive in a variable environment (Venable and Brown 1988; Satterthwaite 2010). Brownsey et al. (2013b) found no evidence for primary dormancy in California populations of *D. graveolen*s, and we found in other germination experiments with California populations that only dead seeds did not germinate under incubation conditions like those reported here. However, germination experiments in the native range showed higher levels of viable ungerminated seeds, closer to 20% (Lustenhouwer et al. 2018). Ongoing studies in our group will provide new insights into seed bank dynamics in the future.

We quantified differentiation between populations in roadside and vegetated habitats using a multi-faceted approach to maximize our chances of observing adaptive differences if there were any. The germination and greenhouse studies under controlled conditions allowed us to minimize other sources of variance and maximize sample size. In contrast, the field study subjected *D. graveolens* plants to realistic environmental conditions with high competition and mortality. Our field site was similar to the vegetated areas where *D. graveolens* is actively invading, including dominant species shared with the vegetated source sites (Tables S4 and S2, respectively). Therefore, we expected that adaptive differences between the source populations should have been revealed under the field conditions. However, it is impossible to eliminate the possibility that adaptive differentiation could be exposed under different environmental conditions.

We looked for population differentiation for plant phenology and did not find any; nor did we find differentiation in the phenology response to stress. Plant competition can lead to physiological stress if resources are limited, and physiological stress can strongly affect plant phenology (Aragón et al. 2007). Competition can initiate stress-induced flowering in some Mediterranean plant species (Takeno 2016). Development time may respond to stress by advancing or delaying reproduction (Fox 1990); such phenotypic plasticity is not necessarily adaptive, but it can be (Anderson et al. 2012). Previous work suggests that flowering time in *D. graveolens* can evolve; plants from the expanding northern edge in Europe flowered earlier in a common garden (Lustenhouwer et al. 2018). Such rapid adaptation in flowering time is commonly seen in response to shifts in latitude in invasive plants (e.g., Leger and Rice 2007; Colautti and Barrett 2013; van Boheemen et al. 2019). Changing phenology can have strong fitness effects on invasive plants (Colautti and Barrett 2013) and may increase competitive effects on other plant species (Alexander and Levine 2019). We did find differences between field treatments affecting time to flowering; initially, some plants began reproducing sooner in the grassland control plots, which could be explained by stress-induced flowering, although overall plants flowered earlier in the competitor-removal plots. While we observed marked phenotypic plasticity in phenology, we did not find evidence for adaptive divergence between roadside and vegetated sites for either phenology or phenotypic plasticity in phenology.

We did not control for the maternal environment of seeds in our study. Environmental variation for field-collected seeds can influence the results of common garden studies with invasive plants (e.g., Turner et al. 2014). Ideally, we would have replicated the entire experiment with a second set of seeds generated in the greenhouse. Unfortunately, we could not delay our experiments (which formed part of a PhD dissertation) for the 12 months required to grow this extra generation. A standard indicator of variability in the quality of the maternal environment is seed size; seed weights in our sample did not show consistent differences between collections from roadside and vegetated sites (Table S4), indicating that overall seed quality was similar. However, the seed germination rate was lower in vegetated sites, which could reflect lower seed viability. We predicted that evolution would result in greater competitive ability in populations in vegetated sites, and our data did not support our prediction. If patterns of maternal provisioning or epigenetics in the vegetated sites systematically reduced survival, biomass, and response to competition relative to roadside sites, then maternal effects could have masked adaptive differentiation.

Some *D. graveolens* populations are spreading away from roadsides and successfully invading plant communities; our results suggest that rather than locally adapted populations, these plants in vegetated communities are able to grow there when they can get a foothold because of phenotypic plasticity. Baker’s (1965) concept of the “general purpose genotype” of colonizing species proposed that phenotypic or developmental plasticity underlies the success of many weedy invaders (Parker et al. 2003). Although we saw that plants of all origins were negatively impacted by competition in the greenhouse (Fig. 4) and in the field (Fig. 5), plants in the field experiment were still able to flower with an adjustment in phenology (Fig. 6). These plants, persisting in low numbers in suboptimal conditions, may be able to take advantage of localized or periodically large disturbances such as fires or intensive management activities involving soil disturbance (Hansen and Clevenger 2005).

The evolution of competitive ability in invasive species has been a significant research focus for nearly 30 years, generally in the context of reallocating resources with escape from specialized natural enemies (Blossey and Nötzold 1995). Many studies have compared traits related to competitive ability between populations from the native and introduced ranges (Bakker and Wilson 2001; van Kleunen and Schmid 2003; Bossdorf et al. 2005; Felker‐Quinn et al. 2013; Yuan et al. 2013; Callaway et al. 2022). Studies exploring the evolution of competitive ability with expansion into new habitats within the introduced range are less common (but see Fletcher et al. 2023). According to life history theory, tradeoffs exist between traits that increase fitness in highly competitive environments and dispersal and reproductive traits that favor a ruderal lifestyle in highly disturbed, more open environments (Grime 1977; Pierce et al. 2017). In invasive species, selection for dispersal and reproduction at the invasion front may lead to declines in competitive ability (Burton et al. 2010). In its native range, *D. graveolens* thrives in disturbed soils and is commonly found along roadsides (Brownsey et al. 2013a). This is common in introduced plants, and in fact, ruderal traits may be selected for as introduced plants spread along transportation corridors. Our results suggest that even strong selection in less disturbed, more competitive environments may not result in the rapid evolution of invasive ability as plants spread away from roads. Opposing selection pressures on roads and away from roads, with gene flow linking close populations, may represent an insurmountable barrier to the evolution of increased competitive ability in invasive plants. To the extent that these barriers to adaptation persist over time, evolution will not represent an urgent threat to management activities or risk assessments.

### Supplementary Information

Below is the link to the electronic supplementary material.

Supplementary file1 (PDF 234 KB)

## Data Availability

Experimental data and code supporting the findings of this study are deposited in Dryad: 10.5061/dryad.wdbrv15wz.
