# Supplementary material for: Invasion away from roadsides was not driven by adaptation to grassland habitats in Dittrichia graveolens (stinkwort)
Source: Biol Invasions. 2024 Jun 5;26(9):2923–39. doi: 10.1007/s10530-024-03359-6 (PMC11319513; doi:10.1007/s10530-024-03359-6)
Supplement: Supplementary file 1 — Supplementary file1 (PDF 234 KB) [file 10530_2024_3359_MOESM1_ESM.pdf]

Table S1 Site characteristics for roadside and vegetated habitats at each of the 8 paired sites in the County of Santa Clara, California

Site	Road Density (km/km2) within 1 km Radius		Elevation (m)		Road Width (m)	Distance Between Habitats (m)	Latitude/Longitude		Substrate Category	
	Roadside	Vegetated	Roadside	Vegetated			Roadside	Vegetated	Roadside	Vegetated
Baylands Park	7.659	6.702	3.05	3.05	34.6	539.72	37°24'50" N 122°0'5" W	37°24'50" N 121°59'43" W	soil	soil
Chesboro Reservoir	3.112	3.151	170.69	164.59	9.2	39.50	37°7'33" N 121°42'26" W	37°7'32" N 121°42'25" W	soil	soil
Guadalupe Reservoir	1.17	1.165	195.07	185.93	9.6	39.48	37°11'52" N 121°52'46" W	37°11'53" N 121°52'45" W	concrete	soil
Lexington Reservoir	4.744	4.495	207.26	210.31	10.1	92.66	37°12'2" N 121°59'9" W	37°11'59" N 121°59'9" W	concrete	rock (natural)
Oakridge Pond	13.03	12.029	57.91	57.91	25.7	252.91	37°15'22" N 121°51'58" W	37°15'16" N 121°52'5" W	soil	soil
Parkway Lakes RV	4.23	4.339	97.54	94.49	10.9	172.26	37°10'43" N 121°41'17" W	37°10'43" N 121°41'24" W	soil	rock (natural)
Penitencia Creek	12.785	12.925	70.10	79.25	9.4	122.70	37°23'38" N 121°50'23" W	37°23'38" N 121°50'18" W	soil	rock (natural)
South San Jose VTA	13.707	13.672	64.01	64.01	15.1	49.19	37°14'13" N 121°47'17" W	37°14'13" N 121°47'15" W	concrete	rock (natural)

Table S2 Resident species growing at each of the 8 paired sites in the County of Santa Clara, California. We surveyed the sites between July 1st and August 14th, 2020

**Populations**	**Origin**	**Baylands Park**		**Chesboro Reservoir**		**Guadalupe Reservoir**		**Lexington Reservoir**		**Oakridge Pond**		**Parkway Lakes RV**		**Penitencia Creek**		**South San Jose VTA**	
**Scientific Name**		Roadside	Vegetated	Roadside	Vegetated	Roadside	Vegetated	Roadside	Vegetated	Roadside	Vegetated	Roadside	Vegetated	Roadside	Vegetated	Roadside	Vegetated
Anagallis arvensis	Non-native				X								X				
Baccharis pilularis	Native				X		X	X	X				X		X		
Brassica nigra	Non-native		X		X			X	X			X		X			
Centaurea solstitialis	Non-native											X	X				
Cyclospermum leptophyllum	Non-native													X			
Cynodon dactylon	Non-native						X										
Epilobium brachycarpum	Native				X		X						X			X	X
Erigeron bonariensis	Non-native	X												X			
Erigeron canadensis	Native	X															
Foeniculum vulgare	Non-native											X					
Genista monspessulana	Non-native							X	X								
Gnaphalium palustre	Native	X			X		X	X	X								
Heterotheca grandiflora	Native												X				
Medicago polymorpha/ arabica	Non-native			X			X				X		X	X			
Melilotus albus/ indicus	Non-native						X		X								
Plantago elongata/ erecta	Native							X				X					
Polycarpon tetraphyllum	Non-native													X			
Polypogon monspeliensis	Non-native				X		X		X								
Sonchus asper/ oleraceus	Non-native					X							X	X			
Spergularia rubra	Non-native			X													
Acmispon sp.	Native																X
Artemisia sp.	Native								X								

**Populations**	**Origin**	**Baylands Park**		**Chesboro Reservoir**		**Guadalupe Reservoir**		**Lexington Reservoir**		**Oakridge Pond**		**Parkway Lakes RV**		**Penitencia Creek**		**South San Jose VTA**	
**Scientific Name**		Roadside	Vegetated	Roadside	Vegetated	Roadside	Vegetated	Roadside	Vegetated	Roadside	Vegetated	Roadside	Vegetated	Roadside	Vegetated	Roadside	Vegetated
Avena sp.	Non-native		X	X									X				X
Bromus sp.	Non-native			X		X				X	X	X		X			X
Erodium sp.	Non-native			X	X						X						
Euphorbia sp.	Non-native							X									
Heliotropium sp.	Native				X												
Malva sp.	Non-native													X			
Polygonum sp.	Non-native						X		X								
Trifolium sp.	Unknown			X													
Verbena sp.	Unknown								X								
Unidentified annual Poaceae #1	Non-native		X														
Unidentified annual Poaceae #2	Non-native		X														
Unidentified annual Poaceae #3	Non-native	X															
Unidentified Brassicaceae	Unknown								X								

Table S3 Plant species growing within the fenced field plot at Blue Oak Ranch Reserve, California, surveyed on May 16, 2021

**Scientific Name**	**Family**	**Abundance**	**Origin**
Amsinckia menziesii var. *intermedia*	Boraginaceae	Rare	Native
Avena barbata	Poaceae	Dominant	Non-native
Bromus diandrus	Poaceae	Common	Non-native
Bromus hordeaceus	Poaceae	Common	Non-native
Bromus madritensis ssp. *rubens*	Poaceae	Common	Non-native
Carduus pycnocephalus	Asteraceae	Uncommon	Non-native
Centaurea solstitialis	Asteraceae	Common	Non-native
Clarkia purpurea ssp. *quadrivulnera*	Onagraceae	Common	Native
Daucus pusillus	Apiaceae	Common	Native
Erodium cicutarium	Geraniaceae	Dominant	Non-native
Festuca myuros	Poaceae	Common	Non-native
Festuca perennis	Poaceae	Common	Non-native
Lupinus bicolor	Fabaceae	Common	Native
Lysimachia arvensis	Primulaceae	Uncommon	Non-native
Madia gracilis	Asteraceae	Rare	Native
Trifolium hirtum	Fabaceae	Dominant	Non-native
Vicia hirsuta	Fabaceae	Uncommon	Non-native

Table S4 We collected seeds from 8 paired populations in the County of Santa Clara, California, and took one homogenized sample of 30 seeds from each population and weighed them to the closest 0.001g. We calculated average seed mass for each source habitat using a Welch Two Sample t-test. Average seed mass varied from 0.243 to 0.333 and did not differ between source habitats (roadside = 2.26 mg, vegetated = 2.37 mg; $t_{12.11} = -1.18$, $P = 0.259$)

**Site Name**	**Average Seed Weight (mg)**	
	Roadside	Vegetated
Baylands Park	0.273	0.277
Chesboro Reservoir	0.267	0.297
Guadalupe Reservoir	0.300	0.283
Lexington Reservoir	0.280	0.243
Oakridge Pond	0.267	0.333
Parkway Lakes RV	0.320	0.307
Penitencia Creek	0.273	0.320
South San Jose VTA	0.277	0.310

To visualize differences in plant community composition across our sites, we used the vegan package[1] in R (R Core Team, 2022) to perform an NMDS analysis on the plant community survey data collected from each paired site (Table S2). When we compared vegetated and roadside habitats, our NMDS analysis revealed overlapping ellipses and clustered points, indicating similarities in plant community composition. Specific plant species, including *Bromus* sp., *Epilobium brachycarpum*, and *Gnaphalium palustre*, correlated with values of the 2 NMDS factors as shown in Figure S1 ($P < 0.001$, $R^2 > 0.422$). We tested for differentiation between roadside and vegetated habitats with PERMANOVA and found no significant overall difference between the habitats using the 'adonis2' function from the vegan package ($F_{1,14} = 1.51$, $P = 0.227$).

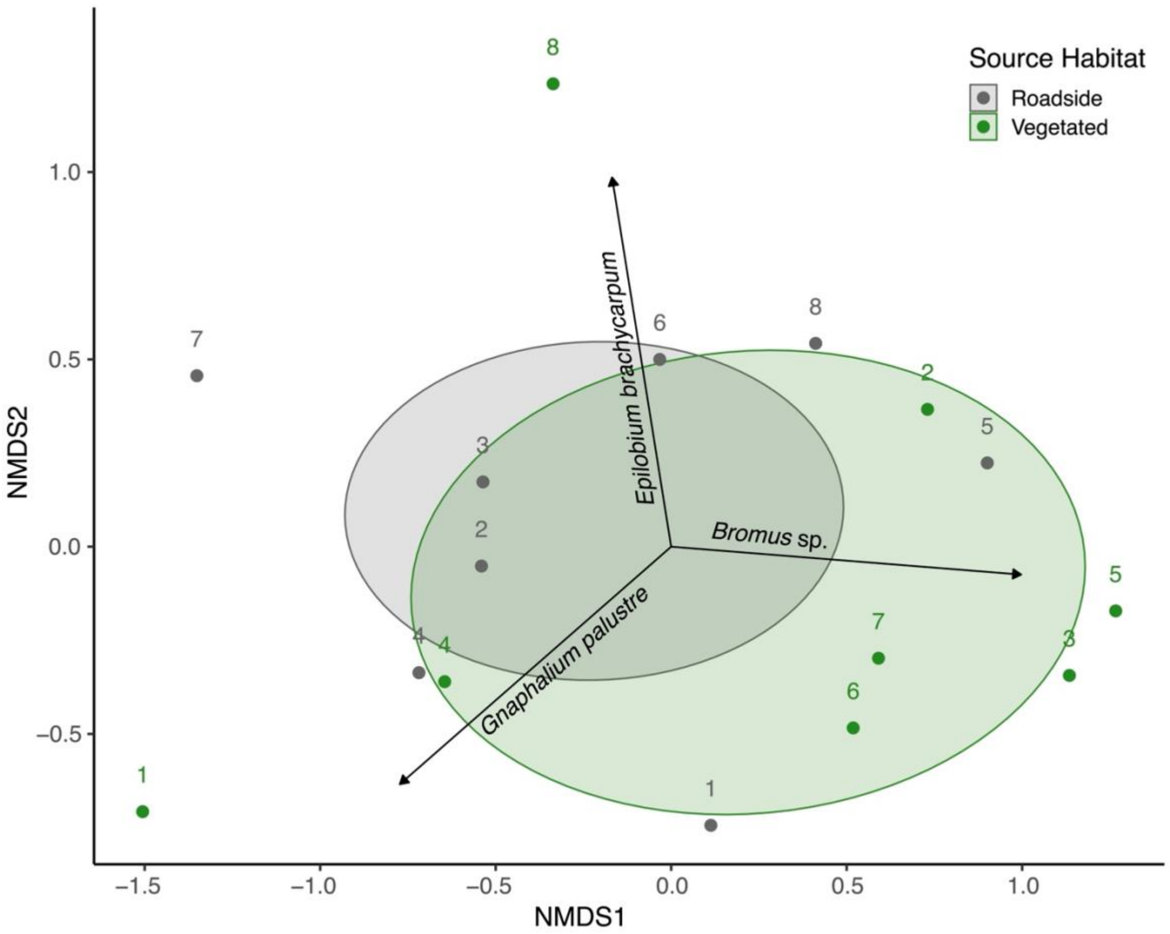

Fig. S1 When we compared vegetated and roadside habitats, our NMDS analysis revealed overlapping ellipses and clustered points, indicating similarities in plant community composition. Specific plant species, including *Bromus* sp., *Epilobium brachycarpum*, and *Gnaphalium palustre*, correlated with values of the 2 NMDS factors. Numbers denote site pairs

[1] Oksanen J, Simpson G, Blanchet F, et al (2022) vegan: Community Ecology Package
